# B cell receptor signaling strength modulates cancer immunity

**DOI:** 10.1172/JCI157665

**Published:** 2022-03-15

**Authors:** Jian Ye, Peter P. Lee

**Affiliations:** Department of Immuno-Oncology, Beckman Research Institute, City of Hope Comprehensive Cancer Center, Duarte, California, USA.

## Abstract

Tumor-infiltrating B cells exert antitumor effects by producing antibodies against tumor-associated antigens. Conversely, B cells may promote tumors through the production of factors that dampen antitumor immunity. In this issue of the *JCI*, Bing Yang, Zhen Zhang, et al. investigated the roles of B cell receptor (BCR) signaling in antitumor immunity, focusing on the role of an Asia-specific variant of human immunoglobulin G1 (IgG1) containing a Gly396 to Arg396 substitution (hIgG1-G396R) in colorectal cancer (CRC). Epidemiological analysis revealed an association between hIgG1-G396R and progression-free survival in CRC. Human samples and mouse models of CRC showed plasma cells, as opposed to B cells, infiltrating the tumor microenvironment. Notably, patients with the hIgG1-G396R variant had increased CD8^+^ T cells, dendritic cells, and tertiary lymphoid structure density. These findings indicate that the hIgG1-G396R variant represses tumorigenesis by enhancing B cell responses, and suggest that modulating BCR signaling could improve the efficacy of immunotherapy in cancer.

## B cells can exert antitumor activity

While current cancer immunotherapeutic strategies focus extensively on augmentation of immune surveillance by T cells, the role of B cells in cancer has only begun to emerge in recent years. Tumor-infiltrating B cells exert both antitumor and protumor effects, depending on their immune-stimulatory or immune-suppressive activities, which vary with cancer type (1, 2). A meta-analysis of 69 studies in 19 cancers showed a positive association of B cell infiltration with favorable prognosis in half of the studies, compared with less than 10% of studies showing a negative association (3). Beyond numbers, spatial patterns of B cells within tumors are important. Even when tumors have similar B cell densities, differing cluster patterns may lead to different clinical outcomes (4).

B cells can exert antitumor activity via production of antibodies against tumor-associated antigens (TAAs). Antibodies in turn can impact downstream immune responses, including antibody-dependent cellular cytotoxicity (ADCC) by NK cells, phagocytosis by macrophages (ADCP), and antigen presentation and T cell priming by dendritic cells (DCs) (1, 2). B cells express MHC II to directly serve as antigen-presenting cells, triggering T cell activation, including that of T follicular helper cells (5–7). As such, B cells are the major component and initiator of tertiary lymphoid structures (TLSs), which are favorable prognostic markers for colorectal, lung, breast, and pancreatic cancers (2). TLSs also associate with improved responses to cancer immunotherapy and survival in melanoma and soft-tissue sarcomas (8–10). Moving beyond the tumor, B cells within sentinel lymph nodes (SLNs) correlate with improved clinical outcome (11).

## B cells and poor clinical outcome in some cancers

On the other hand, B cells have also been correlated with poor clinical outcomes in some cancer types, such as renal cancer carcinoma and glioblastoma (2). Mechanistically, immune-suppressive subsets of B cells, characterized by IL-10 and TGF-β production, can dampen antitumor immunity partially through inhibiting effector T cells and enhancing regulatory T cell induction (12). Excessive production of antibodies by plasma cells can also lead to immune complexes that may modulate the tumor microenvironment (TME) and induce myeloid-derived suppressor cells (2). A recent study provided evidence that B cells can elicit IL-10^+^ macrophages by synthesizing and secreting the neurotransmitter GABA, which in turn can inhibit the cytotoxic function of CD8^+^ cells (13). These studies illustrate the complex and heterogeneous roles of B cells within the TME. Thus, further characterizing how B cell responses are initiated and regulated within the TME (and SLNs) will help to develop clinical strategies for cancer immunotherapy.

Antigen contact with the B cell receptor (BCR) and subsequent signal transduction are the initial steps for B cell activation, proliferation, and subsequent plasma cell differentiation. To investigate the roles of BCR signaling modulation in antitumor immunity, the study in this issue of the *JCI* by Bing Yang, Zhen Zhang, et al. (14) followed up on their recent report on an Asia-specific variant of human immunoglobulin G1 (IgG1) containing a Gly396 to Arg396 substitution (hIgG1-G396R) in colorectal cancer (CRC). In their previous study, the authors demonstrated that hIgG1-G396R homozygous carriers are enriched in cohorts of patients with systemic lupus erythematosus (SLE) (15). Mechanistically, the hIgG1-G396R variant enhances phosphorylation of the IgG1 immunoglobulin tail tyrosine (ITT) motif, which in turn stabilizes binding of the adaptor Grb2 and upregulates downstream Brb2/Bruton’s tyrosine kinase (Btk) signaling, leading to a burst of broad-spectrum autoantibody production. The enhanced B cell activation and IgG1 production contribute to development of SLE. Autoimmunity and tumor immunity may be considered two sides of the same coin (16, 17). In fact, SLE patients are known to have altered risks for different cancers (18, 19). As such, it is reasonable to expect that the hIgG1-G396R variant may also impact cancer risk.

## The hIgG1-G396R variant and CRC

**Figure 1:**
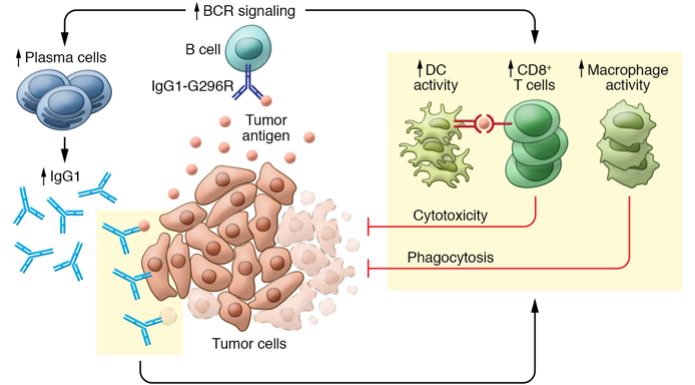
Protective effects of hIgG1-G396R in CRC. In the presence of tumor antigen, B cells expressing the IgG1-G396R variant are activated, differentiate into plasma cells, and produce large quantities of antibodies. IgG1^+^ plasma cells, macrophages, CD8^+^ T cells, and DCs infiltrate the TME to exert antitumor effects.

Bing Yang, Zen Zhang, and colleagues first investigated whether the hIgG1-G396R variant correlated with risk of CRC using epidemiological data. Indeed, hIgG1-G396R was a positive prognostic factor for both overall survival and progression-free survival in CRC. Intriguingly, this variant showed even greater benefit in microsatellite stability (MSS), as opposed to microsatellite instability (MSI), in patients with CRC. To investigate the protective effect, the authors generated a knockin mouse model harboring a hIgG1-G396R homolog (G400R mutation in mIgG2c) that can induce ADCC and ADCP in mice, with binding affinity for FcγRs similar to that in humans. Homozygous mIgG2c-G400R mice had reduced tumor burden in two mouse orthotopic tumor models, as well as in a chemical model in which azoxymethane/dextran sodium sulfate (AOM/DSS) induced colitis-associated carcinoma. These results led the authors to further investigate the mechanism of the protective effect of hIgG1-G396R in CRC (Figure 1). They found elevated plasma cells, but not B cells, infiltrating the TME of both human and mouse CRC. In ovalbumin-immunized mice expressing mIgG2c-G400R, wild-type mIgG2c, or mIgG2c with a truncated cytoplasmic tail (mIgG2c-tailless), in which the BCR signals are sequentially reduced, the authors showed that the variant promoted differentiation of antigen-specific plasma cells and memory B cells. In addition, immunohistology and single-cell sequencing indicated that patients with the hIgG1-G396R variant had increased CD8^+^ T cells, DCs, and TLSs. A similar result was shown in murine tumor models in a BCR signal dose–dependent manner. These findings suggest that the hIgG1-G396R variant might reshape the TME through potentiating B cell activation and promoting the generation of CD8^+^ T cells and DCs into TLSs.

Having established that hIgG1-G396R enhances IgG1-producing plasma cell differentiation, the authors tested whether this variant could potentiate production of tumor-specific IgG1. By using microarrays containing a panel of TAAs, the authors showed increased TAA-specific IgG1 antibodies, but not IgM or other IgG subclasses, in human CRC patients. Consistently, in the IgG2 mouse model, reduction in tumor burden in mice challenged by antigen-expressing cancer cells relied on the strength of the BCR/IgG signal. In addition, the authors found that mice carrying the mIgG2c-G400R variant exhibited increased phagocytic activity in macrophages and upregulated antibody-mediated tumor antigen uptake by DCs, with subsequent antigen-specific T cell activation. These results support the hypothesis that upregulated antitumor activity with this IgG variant is partially due to enhanced ADCP and antigen presentation. The authors performed the important next step, determining whether B cells bearing this variant, or IgG purified from cancer-bearing animals, had potential clinical value. Bing Yang, Zhen Zhang, and colleagues injected tumor-specific memory B cells, or purified IgG from the serum of tumor-bearing mice, into mice inoculated subcutaneously with colon cancer cell lines. Both treatments inhibited tumor growth. Taken together, the mechanistic studies by Bing Yang, Zhen Zhang, et al. indicate that modulation of B cells, especially manipulating BCR/IgG signaling, could have a therapeutic effect in tumors.

## Clinical implications

Reflective of the dual pro- and antitumoral functions of B cells, clinical trials have attempted to deplete or augment B cells. Clinical trials with anti-CD20 antibody for B cell depletion as a therapeutic approach in solid cancers did not show efficacy (20), likely due to the complexity of B cell function and composition in cancer. On the other hand, B cells may be stimulated (such as via CD40) in vivo or adoptively transferred as cellular immunotherapy (21). In the study by Bing Yang, Zhen Zhang, et al., adoptive transfer of tumor-specific memory B cells with increased BCR signaling demonstrated promising antitumor activity (14). As CRC patients with the hIgG1-G396R variant show increased TLS density and TLS area within the TME, it is possible that modulating BCR signaling could improve the efficacy of immune checkpoint inhibitor therapy. While this IgG variant was identified in an Asian population, prevalence of this or similar variants in other populations remains to be determined.

Interactions between T cells and B cells are critical for efficient antitumor immunity (5). Not only are T cells involved in regulating the differentiation and maturation of germinal center B cells, but this interplay is bidirectional such that B cells are needed for the differentiation and functionality of follicular helper T (Tfh) cells. Mice with B cell deficiency or immunized with tumor antigen lacking the BCR-specific epitope failed to generate Tfh cells, and subsequently did not develop effective antitumor immunity (5). Based on emerging data on the importance of B cells in antitumor immunity, development of monotherapy or combination therapy modulating B cells and/or BCR signaling could lead to effective cancer immunotherapies.

## Version 1 03/15/2022

Electronic publication
